# A Further Thyroid "Discovery."

**Published:** 1920-03-13

**Authors:** 


### THE CONTROL OF ANIMAL GROWTH.

### A Further Thyroid "Discovery."

It was but a short time ago that we had opportunity to call attention to some remarkable flights of journalistic fancy upon the subject of old age and the thyroid gland. Momentarily so apparently interesting, and yet of such very sudden ending, this so-called attempt at public education was peculiarly similar to others of its class, and as another scientific wonder in the Press was already overdue, it was, perhaps, not surprising, a week or so ago, to read an outburst of applause for a brand-new discovery. What might well have caused surprise, however, was that the thyroid gland should again be singled out for attention, and once more the marvellous possibilities of thyroid administration to the weakened human body be boomed throughout the country. It is hard to imagine whether it was the catch-word "rejuvenation," or the instinctive attraction towards mysterious creatures habitually lurking in darkness and water, that was intended to draw the public attention. In any case, extraordinarily little seems to have been achieved in the educative sense, whereas a state of yet further confusion was inevitably produced.

Thinking over this remarkable outburst, the memory of an article by Mr. Julian S. Huxley himself, published in the *Sphere* of February 28, came as something of an antidote. It was written by a biologist and largely from a biologist's standpoint, but it did explain the situation, and if the author be forgiven for leading along many a fascinating line of thought, only abruptly to leave the reader stranded when his facts gave out, there is little fault to find with his proofs that thyroid gland activity is found to exert considerable control over the growth and metamorphoses of certain amphibians.

### The Medical Attitude.

He sums up the medical attitude, however, and, indeed, the present state of not only medical but also biological and physiological knowledge—as applied to practical treatment of the human race, when he says: "So if there is too little, they (the doctors) make up the balance by supplies from without; if there is too much, they operate and remove the excess." Indeed, if we may subscribe a medical opinion on thyroid therapy as a whole, where the restrictions are imposed by the facts that humans and not tadpoles are to be treated, it seems that there is not only remarkably little possibility of doing anything else in "deficiency" conditions, even if a more exact active principle than "extract" be invented, but also with regard to "excess" conditions we must hope for an "anti-thyroid principle" or go on as we are going and try somehow to reduce the gland's activity. Medically speaking, the tadpole experiments are almost exactly paralleled by the instances of human cretins, etc., which have long been recognised as conditions dependent upon the degree of thyroid activity.

Let us, however, appreciate while criticising. The true point of these experiments is that they show physiologists to be on the verge of discovering how to turn on and off the switch which controls the rate and, to some extent, the form of animal growth. When completed the possibilities arising from such a discovery would be manifold, but we certainly hesitate to jump to the conclusion that speeding and livening up the metabolism of an animal organism will cause an increase in the length and utility of its life. The simple reasoning of a mere medical man suggests that, though for a time a more active, perhaps larger, creature will exist, there will be a proportionate decrease in the natural span of its life. Which type would, in the long run, provide the more useful race and more productive community is a question needing far deeper consideration and having many more side issues than some of our lay contemporaries would appear to realise. The question of thyroid and rejuvenation and the ridiculous muddle made between this relationship and the testicular grafts of M. Voronoff we have already dealt with in these pages (October 25, 1919, p. 71), and the subject does not call for a second comment.

### Our Present Measures.

To those, therefore, who would read a concise and well-written sketch of biological activities in the thyroid question we may heartily recommend Mr. Huxley's article, but, granting the physiological proofs, since the medical situation remains restricted by the necessity of dealing with human sufferers as they are met, we will note briefly a practical medical conclusion recently published. Direct action of the gland extract in cases of cretinism and myxœdema is now so well recognised and so often experienced that we may pass directly to exophthalmic goitre. The prognosis in this type of case is of great importance to the practitioner, for the patient, already nervous, is insistent upon knowing the future course of his trouble. Some cases, well seen in certain war types, get well almost without treatment, and radiotherapy can claim to produce with milder instances a considerable percentage recovery within three months. If, however, the tendency is for the gland and symptoms to increase, then probably operative removal of part of the gland is the only satisfactory procedure. In practice with modern surgical technique it is only a small residue of cases which then go from bad to worse, but these must be allowed for in a spoken prognosis. They may finally develop sugar and albumen in the urine, an intermittent pulse from myocardial degeneration, and all the well-known symptoms of hyperthyroidism. Perhaps if we were generally more complete in our removals this residue of cases would be still further reduced, and in any case records from the Mayo clinic suggest that subtotal thyroidectomy should be performed,

**The Control of Animal Growth**—(*continued*).

and that a far more extensive operation than the removal of one lobe is nearly always advisable.

This suggestion is at least in accord with the results of past as well as recent animal experiment, but let us remember that the over-production of thyroid principle in goitre is an abnormal, a pathological condition, and its *cause* is a quite separate consideration. It is a great pity, apart from the fact of how little medical or any other science really knows concerning the exact functions of either the normal or diseased thyroid gland, that such matters as the recent animal experiments cannot be presented to the public, as indeed Mr. Huxley himself does, simply as valuable links in a long chain of research.

